# Erosive potential of vitamin waters, herbal drinks, carbonated soft drinks, and fruit juices on human teeth: An in vitro investigation

**DOI:** 10.34172/joddd.2023.40413

**Published:** 2023-11-11

**Authors:** Rudee Surarit, Kanonrat Jiradethprapai, Kanyakorn Lertsatira, Jarukan Chanthongthiti, Chayada Teanchai, Sivaporn Horsophonphong

**Affiliations:** ^1^Department of Oral Biology, Faculty of Dentistry, Mahidol University, Bangkok, Thailand; ^2^Faculty of Dentistry, Siam University, Bangkok, Thailand; ^3^Undergraduate Program, Faculty of Dentistry, Mahidol University, Bangkok, Thailand; ^4^Research Office, Faculty of Dentistry, Mahidol University, Bangkok, Thailand; ^5^Department of Pediatric Dentistry, Faculty of Dentistry, Mahidol University, Bangkok, Thailand

**Keywords:** Beverages, Carbonated beverages, Fruit juices, Herbals, Tooth erosion, Vitamins

## Abstract

**Background.:**

Dental erosion is the loss of dental hard tissues through the acid dissolution of tooth minerals. One of the major factors that cause erosion is the consumption of acidic food and drinks. This study investigated and compared the effect of vitamin waters, herbal beverages, carbonated soft drinks, and fruit juices on the loss of human dental hard tissue.

**Methods.:**

Human tooth samples were immersed in various drinks: vitamin waters, herbal beverages, carbonated soft drinks, and fruit juices. The pH value of each drink was measured using a pH meter. The weight of each sample was determined before and after six days of immersion in the tested drink, and the weight loss percentage was calculated. The exposed tooth surfaces were also examined under a scanning electron microscope.

**Results.:**

Most of the tested drinks were acidic and displayed pH values lower than the critical pH for enamel erosion. Significant weight loss of the tooth samples was found in all tested drink groups. Additionally, the samples immersed in fruit juices and herbal beverages exhibited significantly greater weight loss than those immersed in carbonated soft drinks. Scanning electron micrographs showed samples immersed in the tested drinks to demonstrate structural disintegration with occasional void spaces, except for samples immersed in Doi Kham^®^ Lemongrass drink.

**Conclusion.:**

Most of the tested drinks have the potential to cause dissolution and destruction of dental hard tissues. Consumers should be aware that prolonged exposure to these drinks could lead to permanent loss of tooth mineral and dental erosion.

## Introduction

 Dental erosion is the loss of dental hard tissues through the acid dissolution of tooth minerals without the involvement of acid from bacterial origin.^1^ Loss of minerals causes permanent damage to the tooth structure, leading to tooth sensitivity, dimensional changes, caries susceptibility, pulpitis, and permanent tooth loss.^2^ Dental erosion has increased in prevalence and severity and has become a significant problem in recent years.^3-6^ It affects individuals in all age groups; signs and symptoms of dental erosion have been reported in children, adolescents, adults, and older people.^4,5,7,8^

 One of the major causes of dental erosion is the consumption of acidic foods and beverages.^2,3^ The popular beverages known to be acidic are carbonated soft drinks and fruit juices,^9,10^ which have been demonstrated to cause dental erosion in populations worldwide.^3-5,11^

 Nowadays, people are conscious about their health, which has led to changes in beverage consumption trends. In particular, functional drinks such as vitamin waters and herbal beverages have gained increasing popularity in recent years.^12,13^ However, while vitamin waters and herbal beverages are promoted as having health benefits due to their ingredients consisting of vitamins, antioxidants, and herbal ingredients with natural origin and are good for physical health,^14^ the erosive effects of these drinks on human teeth are rarely mentioned.^15-17^ Therefore, we aimed to investigate and compare the erosive impact of vitamin waters, herbal beverages, carbonated soft drinks, and fruit juices on human dental hard tissue.

## Methods

 This study investigated four commercially available drinks: vitamin waters, herbal beverages, carbonated soft drinks, and fruit juices. Each group consisted of four commercial products, totaling 16; these popular and commercially available products were brought off-the-shelf at supermarkets in Bangkok, Thailand. Table 1 presents the details of the drink products, including their major ingredients and manufacturers. Lime juice and distilled water served as the positive and negative controls, respectively.

**Table 1:** pH values of the selected drinks

**Drink type**	**Product**	**pH value of the product**	**pH value of the group**
		**Mean (SD)**	**Median (min/max)**
Vitamin waters	1) VITADAY^®^: General Beverage Co., Ltd., Nakhon Pathom, Thailand	3.42 (0.006)	3.42 (3.13/3.68)
	2) Mansome^®^: T.C. Pharmaceutical Industries Co., Ltd., Bangkok, Thailand	3.40(0.040)	
	3) B’lue^®^: Danone Sappe Beverages Co., Ltd., Bangkok, Thailand	3.18(0.046)	
	4) C-vitt^®^: Coca-Cola Co., Ltd., Bangkok, Thailand	3.64(0.045)	
Fruit juices	1) Tipco^®^ Tangerine juice: Tipco Foods Public Co., Ltd., Bangkok, Thailand	3.62 (0.006)	3.76 (3.61/4.17)
	2) Malee^®^ Pineapple juice: Malee Group Public Co., Ltd., Pathumthani, Thailand	3.76 (0.006)	
	3) Doi Kham^®^ Tomato juice: Doi Kham Co., Ltd., Bangkok, Thailand	4.15 (0.015)	
	4) Tipco^®^ Apple juice: Tipco Foods Public Co., Ltd., Bangkok, Thailand	3.75 (0.031)	
Carbonated soft drinks	1) Coca-Cola^®^: Coca-Cola Co., Ltd., Bangkok, Thailand	2.62 (0.081)	3.00 (2.56/3.50)
	2) Pepsi^®^: Suntory PepsiCo Beverage Co., Ltd., Bangkok, Thailand	2.63 (0.021)	
	3) Sprite^®^: Coca-Cola Co., Ltd., Bangkok, Thailand	3.48 (0.015)	
	4) Fanta^®^ Red soda strawberry flavor: Coca-Cola Co., Ltd., Bangkok, Thailand	3.30 (0.015)	
Herbal drinks	1) Doi Kham^®^ Roselle: Doi Kham Co., Ltd., Bangkok, Thailand	2.75 (0.03)	3.42 (2.72/5.58)
	2) Tipco^®^ Krachaikhao: Tipco Foods Public Co., Ltd., Bangkok, Thailand	3.55 (0.023)	
	3) QminC^®^ Curcumin: Tera Food & Beverage Co., Ltd., Nakhon Ratchasima, Thailand	3.28 (0.015)	
	4) Doi Kham^®^ Lemongrass with ginger and pandan: Doi Kham Co., Ltd., Bangkok, Thailand	5.56 (0.020)	

###  Estimation of pH 

 A pH meter (3-Star Benchtop pH Meter; Orion^TM^, Huston, Tx, USA) was used to evaluate the pH of each drink product. Three packages of each product with different batch numbers were chosen. Each package was tested before the expiry date indicated on the label, and its pH value was measured in triplicate.

###  Sample preparation

 The dental hard tissue samples in this study comprised human permanent upper premolars extracted for orthodontic reasons. The teeth were caries-free and without any macroscopic defects. The extracted teeth were stored in 0.1% thymol solution and then disinfected and cleaned with 5% NaOCl for 30 minutes. The buccal and lingual enamel walls of the teeth were sectioned using a low-speed water-cooled saw (IsoMet; Buehler, Lake Bluff, IL, USA) and polished with sandpaper. Each enamel sample was about 4.0 × 4.0 × 2.0 mm. The dimensions of the samples were measured using a veneer caliper with a precision of 0.01 mm. Subsequently, the samples were stored in distilled water.

###  Exposure of tooth samples to the tested drinks and determination of weight loss

 This study used the gravimetric method to determine the erosive potential of the drinks by measuring the weight loss of dental hard tissues.^18-20^ This part of the experimental protocol was adopted from the previous reports of von Fraunhofer & Rogers^18^ and Zimmer et al.^20^ A total of 108 enamel samples were prepared and randomly assigned to the 16 drink products and two control drinks (6 enamel samples for each drink product). The samples were weighed before and after immersion using an analytical balance (Mettler Toledo, Columbus, Ohio, USA) with an accuracy of 0.01 mg.

 After determining the initial weight, each sample was placed in a plastic container containing 0.2 mL of the assigned drink sample and kept at 37 °C for six days. On day three of the experiment, the drink samples were refreshed with a new liquid. After six days of immersion, the samples were removed, blotted dry, placed in a desiccation chamber for 30 minutes at room temperature to finish drying, and weighed to determine the final mass.

###  Scanning electron microscopy (SEM)

 The samples were washed with distilled water, cleaned and dried with an ultrasonic cleanser (Sonorex Digitec; Bandelin, Berlin, Germany), and gold-sputtered (SC7620; Quorum, West Sussex, UK). Images of the enamel surface of the samples were monitored and recorded using a scanning electron microscope (JSM-6610LV; JEOL Ltd., Tokyo, Japan) which scanned at an acceleration voltage of 15 kV using a 5-μm and a 10-μm aperture.

###  Statistical analysis

**Figure 1:**
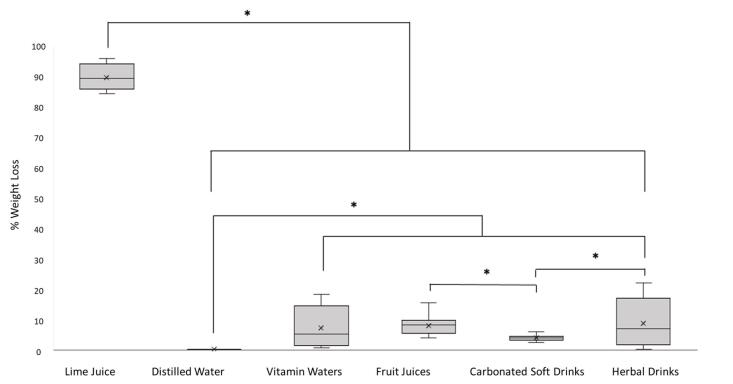
The weight loss percentages of different types of drinks. Straight horizontal lines (⎯) represent medians, and crosses ( × ) represent means (*P < 0.05)

 The pH value of each drink product, including lime juice (positive control) and distilled water (negative control), was reported as the mean ± SD. The summary of the pH value of each drink type was reported as the median (min/max) (Table 1). The weight loss percentage of samples immersed in each drink product was reported as mean (SD) (Table 2), while the weight loss percentage of each type of drink was presented as mean and median (Figure 1).

**Table 2:** Weight of the samples and weight loss percentage

**Groups and products**	**Weight of the samples (g)**			**% Weight loss** **(SD)**
	**Day 0: Before experiment (SD)**	**Day 6: After experiment** **(SD)**	**Weight loss** **(SD)**	
Lime juice (Positive control)	0.07577 (0.00218)	0.00795 (0.00363)	0.06782 (0.00149)	89.616 (4.500)
Distilled water (Negative control)	0.07544 (0.00263)	0.07539 (0.00261)	0.00008 (0.00004)	0.108 (0.053)
**Vitamin waters**				
VITADAY^®^	0.07528 (0.00140)	0.07472 (0.00142)	0.00056 (0.0001)	0.744 (0.135)
Mansome^®^	0.07400 (0.00202)	0.06821 (0.00224)	0.00579 (0.00036)	7.839 (0.624)
B’lue^®^	0.07520 (0.00073)	0.07291 (0.00062)	0.0023 (0.00018)	3.050 (0.217)
C-vitt^®^	0.07519 (0.00272)	0.06244 (0.00232)	0.01275 (0.00073)	16.957 (0.730)
**Fruit juices**				
Tipco^®^ Tangerine juice	0.07376 (0.00255)	0.06636 (0.00296)	0.0074 (0.00292)	9.990 (3.848)
Malee^®^ Pineapple juice	0.07400 (0.00281)	0.06769 (0.00333)	0.00631 (0.00104)	8.550 (1.575)
Doi Kham^®^ Tomato juice	0.07345 (0.00151)	0.06993 (0.00199)	0.00352 (0.00086)	4.799 (1.213)
Tipco^®^ Apple juice	0.07540 (0.00125)	0.06899 (0.00154)	0.00641 (0.00086)	8.497 (1.148)
**Carbonated drinks**				
Coca Cola^®^	0.07564 (0.00189)	0.07331 (0.00182)	0.00234 (0.00031)	3.085 (0.40205)
Pepsi^®^	0.07344 (0.00330)	0.06984 (0.00334)	0.0036 (0.00044)	4.904 (0.63989)
Sprite^®^	0.07445 (0.00211)	0.07141 (0.00241)	0.00304 (0.00044)	4.092 (0.65774)
Fanta^®^ Red soda strawberry flavor	0.07385 (0.00213)	0.07127 (0.00233)	0.00257 (0.0004)	3.492 (0.58972)
**Herbal drinks**				
Doi Kham^®^ Roselle	0.07515 (0.00104)	0.07019 (0.00122)	0.00495 (0.00032)	6.592 (0.48209)
Tipco^®^ Krachaikhao	0.07452 (0.00277)	0.06905 (0.00327)	0.00548 (0.00077)	7.375 (1.22074)
QminC^®^ Curcumin	0.07431 (0.00162)	0.05873 (0.00136)	0.01558 (0.00071)	20.961 (0.81718)
Doi Kham^®^ Lemongrass with ginger and pandan	0.07383 (0.00272)	0.07372 (0.00274)	0.0001 (0.00003)	0.141 (0.04100)

 ANOVA with post hoc Tukey tests were used to compare the initial weights of the samples immersed in each product. The Kruskal-Wallis test, followed by pairwise comparisons, was used to compare the weight loss percentages of immersed samples between different types of drinks. Statistical analysis was performed using the SPSS 25.0 (IBM Corp., Armonk, NY, USA), and a *P* < 0.05 was considered to indicate a significant difference.

## Results

 The pH of each drink product of each group is listed in Table 1. The 16 tested products ranged in pH from 2.62 to 5.56; meanwhile, the pH of lime juice (positive control) was 2.10 ± 0.07, and that of distilled water (negative control) was 5.49 ± 0.19. The median pH value of each drink group ranged from 3.00 to 3.76. Vitamin waters and fruit juices displayed the narrowest pH range, while herbal drinks displayed the widest range.

 The weights of the samples before and after immersion in each drink product and the weight loss percentages are presented in Table 2. There were no significant differences in the weight before immersion (*P* > 0.05). There were significant differences in weight loss percentages between the groups, as illustrated in Figure 1. The samples immersed in lime juice, which served as a positive control, displayed the highest weight loss percentage and significantly differed from other drink types. Conversely, no weight loss was seen in samples immersed in distilled water, i.e., the negative control. All the samples immersed in the tested drinks, including vitamin waters, herbal beverages, carbonated soft drinks, and fruit juices, showed significant weight loss compared to samples in distilled water. Additionally, the samples immersed in fruit juices and herbal beverages had a significantly greater weight loss percentage than those immersed in carbonated soft drinks (Figure 1).

**Figure 2:**
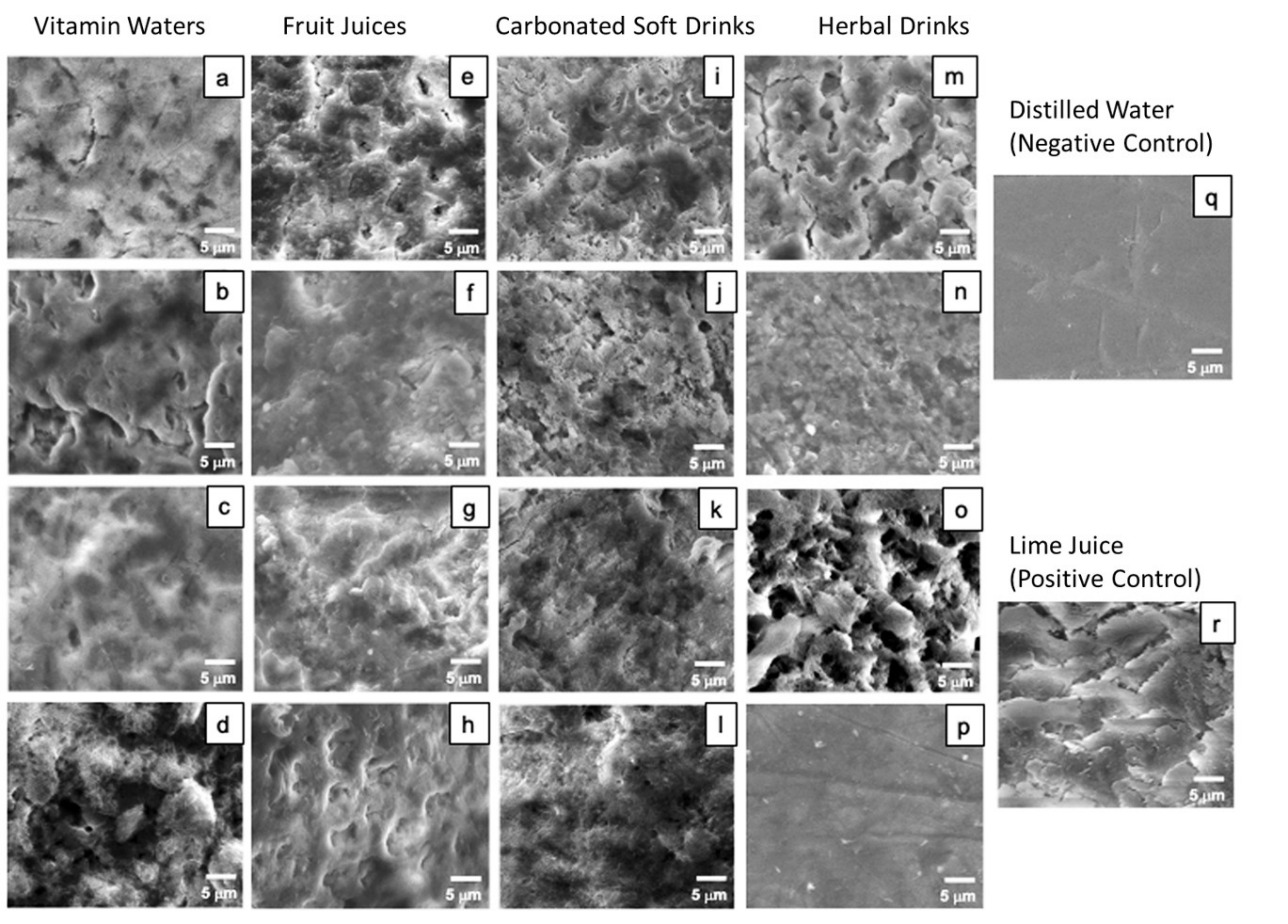
SEM micrographs of sample enamel surfaces after exposure to the tested drinks. a = VITADAY^®^, b = Mansome^®^, c = B’lue^®^, d = C-vitt^®^, e = Tipco^®^ Tangerine juice, f = Malee^®^ Pineapple juice, g = Doi Kham^®^ Tomato juice, h = Tipco^®^ Apple juice, i = Coca Cola^®^, j = Pepsi^®^, k = Sprite^®^, l = Fanta^®^ Red soda strawberry flavor, m = Doi Kham^®^ Roselle, n = Tipco^®^ Krachaikhao, o = QminC^®^ Curcumin, p = Doi Kham^®^ Lemongrass with ginger and pandan, q = Distilled water, r = Lime juice (original magnification × 2000) (Scale bar = 5 µm)

 SEM images were taken after the six-day immersion period (Figure 2). In the distilled water group (negative control), tooth samples displayed normal, intact enamel structure with multiple horizontal lines representing scratches from the sandpaper used to polish the surfaces. Conversely, in the lime juice group (positive control), the samples exhibited severe destruction of the enamel surface. In general, all the samples exposed to the tested drinks exhibited enamel structural disintegration with occasional void spaces. The exceptions were samples immersed in Doi Kham^®^ lemongrass with ginger and pandan, which rarely exhibited any structural change compared to samples immersed in distilled water.

## Discussion

 Excessive acidic beverage consumption can lead to dental erosion.^2,21^ The consumption of carbonated soft drinks was highly associated with the development of dental erosion.^22-24^ A study on the beverage consumption patterns of individuals found a relationship between erosive tooth wear and the frequency of soft drinks, fruit juices, energy drinks, and sports drinks consumption.^25^ Reddy et al^26^ studied the pH of commercially available drinks and discovered that most were acidic, with pH values below 4. Furthermore, the average pH of sodas and fruit juices was around 3, considered highly erosive.^26^ Commercially available vitamin waters were observed to have pH levels of 3‒4, indicating their erosive potential.^27^

 This study investigated the effects of commercially available popular drinks, specifically vitamin waters, herbal beverages, carbonated soft drinks, and fruit juices, on the loss and dissolution of dental mineral tissue, termed dental erosion.^1,2^ Consumption of acidic drinks and beverages is one of the major causes of dental erosion.^2^ In this study, all tested drinks were acidic, with pH values equal to or below the critical pH for enamel dissolution (pH < 5.5). Doi Kham^®^ lemongrass with ginger and pandan featured the highest pH value, equal to the critical pH for enamel dissolution.^28^ Most of the other products displayed low pH values of around 2‒4, which includes values considered to be erosive and extremely erosive.^26^ These low pH values are attributable to acids added to these drinks. For carbonated soft drinks, phosphoric acid is added for tartness, taste, and inhibition of bacterial growth,^26,29^ while carbonic acid is created by carbon dioxide gas infused into water.^29^ Citric and malic acids are added to many vitamin waters, fruit juices, and herbal beverages to give them a naturally strong and sharp flavor.^30,31^ Also, some of the fruits or herbs that were the main ingredients, such as tangerine, pineapple, tomato, apple, and roselle, are acidic in nature.^32-36^ Finally, most vitamin waters contain vitamin C or ascorbic acid, which is acidic.^37^

 This study assessed dental erosion using the gravimetric method, which analyzed the weight loss percentage of dental hard tissues.^18-20^ According to the results, all types of tested drinks, which were vitamin waters, herbal beverages, carbonated soft drinks, and fruit juices, are acidic and can cause significant loss of tooth minerals. The findings are consistent with previous studies that found high acidity of carbonated soft drinks and fruit juices and reported that prolonged exposure to these drinks leads to the dissolution and loss of dental hard tissues.^20,35,38^ Additionally, a few studies have reported that most commercially available vitamin water products are acidic,^17,26,27^ similar to our results, which identified the tested vitamin waters and herbal beverages as acidic.

 In the beginning, before immersion in the tested drinks, the samples showed no significant differences in weight, which implied that the baseline weights of the samples were similar. Significant weight loss was observed after exposure to all groups of tested drinks. Moreover, samples immersed in fruit juices and herbal beverages displayed significantly greater weight loss percentages than those immersed in carbonated soft drinks, consistent with a previous study that found the amount of dental hard tissue loss to be higher in samples immersed in apple and orange juices than those immersed in Coca-Cola^®^.^20^ This may be due to some commercially available carbonated soft drinks containing moderate to high calcium and phosphate levels, resulting in a high degree of saturation which slows down the dissolution of tooth minerals.^9,39,40^

 The dissolution of dental hard tissue by the tested drinks was also confirmed by SEM observations. Specifically, the SEM micrographs revealed structure disintegration and destruction of enamel surfaces, consistent with the weight loss percentages of the samples after immersion. The only drink for which the samples showed no signs of erosion was Doi Kham^®^ lemongrass with ginger and pandan, consistent with the final weights of those samples.

 In this laboratory investigation, tooth samples were exposed to the tested drinks for a long time; therefore, tooth mineral loss may be overestimated relative to the amount lost in the oral cavity. Nevertheless, the findings of this experiment strongly suggest that all types of tested drinks have erosive potential. Specifically, this experiment revealed that when dental hard tissue is exposed to these drinks, the dissolution of tooth minerals does occur. Additionally, the SEM images also confirmed the destruction and erosion of enamel surfaces.

 While vitamin waters and herbal drinks have been marketed as functional drinks for health and have increasingly gained popularity, the impact of these products on dental erosion has rarely been investigated.^15-17,41^ This study showed the erosive potential of vitamin waters and herbal beverages, indicating that long-term exposure to these drinks can cause dissolution and loss of tooth minerals. This study found that most of the tested drinks were acidic; moreover, it illustrated the erosive potential of vitamin waters, herbal drinks, carbonated soft drinks, and fruit juices. Therefore, oral health professionals and consumers should be aware of the potential harm of these drinks.

## Conclusion

 All the tested drinks, which contain vitamin waters, fruit juices, carbonated soft drinks, and herbal drinks, have erosive potential that can cause the destruction and dissolution of dental hard tissues. Moreover, on average, the tested herbal beverages and fruit juices resulted in greater loss of dental hard tissues than carbonated soft drinks. Oral health professionals and consumers should be aware that prolonged exposure to these drinks could permanently damage the tooth mineral structures, resulting in dental erosion.

## Competing Interests

 Authors have no conflict of interest to declare.

## Ethical Approval

 The ethics of this study was approved by the Institutional Review Board (COE.No.MU-DT/PY-IRB 2022/053.0711) of the Faculty of Dentistry/Faculty of Pharmacy, Mahidol University.
